# Bayesian Methods for Multivariate Modeling of Pleiotropic SNP Associations and Genetic Risk Prediction

**DOI:** 10.3389/fgene.2012.00176

**Published:** 2012-09-11

**Authors:** Stephen W. Hartley, Stefano Monti, Ching-Ti Liu, Martin H. Steinberg, Paola Sebastiani

**Affiliations:** ^1^Department of Biostatistics, Boston University School of Public HealthBoston, MA, USA; ^2^Department of Medicine, Boston University School of MedicineBoston, MA, USA

**Keywords:** pleiotropy, SNP, GWAS, prediction, Bayesian

## Abstract

Genome-wide association studies (GWAS) have identified numerous associations between genetic loci and individual phenotypes; however, relatively few GWAS have attempted to detect pleiotropic associations, in which loci are simultaneously associated with multiple distinct phenotypes. We show that pleiotropic associations can be directly modeled via the construction of simple Bayesian networks, and that these models can be applied to produce single or ensembles of Bayesian classifiers that leverage pleiotropy to improve genetic risk prediction. The proposed method includes two phases: (1) Bayesian model comparison, to identify Single-Nucleotide Polymorphisms (SNPs) associated with one or more traits; and (2) cross-validation feature selection, in which a final set of SNPs is selected to optimize prediction. To demonstrate the capabilities and limitations of the method, a total of 1600 case-control GWAS datasets with two dichotomous phenotypes were simulated under 16 scenarios, varying the association strengths of causal SNPs, the size of the discovery sets, the balance between cases and controls, and the number of pleiotropic causal SNPs. Across the 16 scenarios, prediction accuracy varied from 90 to 50%. In the 14 scenarios that included pleiotropically associated SNPs, the pleiotropic model search and prediction methods consistently outperformed the naive model search and prediction. In the two scenarios in which there were no true pleiotropic SNPs, the differences between the pleiotropic and naive model searches were minimal. To further evaluate the method on real data, a discovery set of 1071 sickle cell disease (SCD) patients was used to search for pleiotropic associations between cerebral vascular accidents and fetal hemoglobin level. Classification was performed on a smaller validation set of 352 SCD patients, and showed that the inclusion of pleiotropic SNPs may slightly improve prediction, although the difference was not statistically significant. The proposed method is robust, computationally efficient, and provides a powerful new approach for detecting and modeling pleiotropic disease loci.

## Introduction

Genome-wide association studies (GWAS) have identified numerous single associations between genetic loci and individual phenotypes; but, relatively few GWAS have attempted to detect pleiotropic associations, in which loci are simultaneously associated with multiple distinct phenotypes (Huang et al., 2010, 2011; Zhernakova et al., 2011). However, pleiotropic loci have been inferred and/or identified by various means, both in humans and in model organisms (Chavali et al., 2010; Huang et al., 2010, 2011; Stearns, 2010; Kochunov et al., 2011; Tesse et al., 2011; Zhernakova et al., 2011). These works generally identified pleiotropic candidate loci by identifying overlapping Single-Nucleotide Polymorphisms (SNPs) between two independently run analyses on the same dataset, via meta-analyses of multiple studies, or via ANCOVA (Gupta et al., 2011; Huang et al., 2011; Zhernakova et al., 2011). Statistical methods for joint modeling of multivariate response could be used to capture pleiotropic associations. Several suitable methods have been recently reviewed in Shriner (2012) but they do not seem to be commonly used in statistical genetics (Shriner, 2012).

There are several potential advantages to the direct modeling of pleiotropic associations. First, a model search for loci that are simultaneously associated with multiple phenotypes would likely have higher power than a model search that only considers each phenotype individually. Secondly, more exact modeling may yield more accurate prediction of either or both phenotypes. Thirdly, pleiotropic genes may tend to have a more central role in the relevant functional pathways (Chavali et al., 2010).

Bayesian model search is flexible, robust, and computationally efficient, and lends itself naturally to the creation of genetic risk classifiers. Bayesian classifiers have been used before in GWAS, but generally only on individual phenotypes (McKinney et al., 2006; Sebastiani et al., 2008a,b, 2012a; Okser et al., 2010; Jiang et al., 2011). We recently showed that Bayesian classifiers produce classification rules that are equivalent to using logistic regression with a genetic risk score, and we argued that the advantage of the model approach based on Bayesian classifiers is that it can be generalized to include multiple traits, and gene-gene or gene-environment interaction models (Sebastiani et al., 2012b). We will show that pleiotropic associations can be directly modeled via the construction of simple Bayesian networks, and that these models can be applied to produce Bayesian classifiers, or ensembles of Bayesian classifiers, that leverage pleiotropy to improve genetic risk prediction.

The proposed method includes two phases: (1) discovery of SNPs that could be used for prediction using a Bayesian-model-based approach, and (2) selection of a final set of the most predictive SNPs using cross-validation. In the first phase, Bayesian model comparison is used to determine the most likely disease associations and inheritance modes for each SNP, and then SNPs are ranked by the posterior probability of the association(s). Bayesian classifiers can then be constructed using these SNPs to predict phenotype status either given the genotype data alone or given the genotype data combined with any known phenotype values, if available. In the second phase, we conduct cross-validation to estimate the optimal feature set, so as to avoid over-fitting the mode or, alternatively, applying overly stringent inclusion thresholds. The full details can be found in the methods section.

## Results

### Simulation overview

In total, 1600 GWAS were simulated, with 100 replications each for 16 scenarios. All simulated studies assumed two phenotypes, *D*_A_ and *D*_B_. For each subject, genotype data were simulated for a hypothetical 500,150-SNP assay, and all SNPs were simulated independently. Each scenario specified the number of subjects in the discovery set and their phenotype values, as well as the number of causal SNPs of each type and the range of association strengths for those SNPs. For each simulated GWAS, the exact parameters of each causal SNP (odds ratio, minor allele frequency, mode of inheritance, and disease allele) were randomly selected (See “Methods”). Then the discovery set and 4000-subject validation set were generated using these parameters. More details on the simulation methods can be found in the methods section.

Four distinct sets of simulations were run.

1. Set 1. The first set of simulations tested the algorithms assuming the GWAS were balanced case-control studies of various sample sizes and genetic association strengths, and in which pleiotropy did exist between the two phenotypes. One hundred simulated GWAS were run under each of six scenarios described in Table 1. In all these simulations, there were 150 causal SNPs: 50 associated only with *D*_A_, 50 associated only with *D*_B_, and 50 pleiotropic loci associated with both.
2. Set 2. Like the first simulation set, the second set consisted of 100 simulated GWAS for each of six scenarios. Unlike the first set, the primary phenotype of interest, *D*_a_, was not equally balanced between cases and controls. Instead, only 10% of the subjects in both the discovery and replication sets were “cases” for *D*_a_, whereas cases and controls were balanced for the secondary phenotype, *D*_b_. Further, in all six scenarios the discovery sets consisted of 1000 subjects. As in set 1, the validation set consisted of 4000 subjects (Table 2). The six scenarios varied by the strengths of association for *D*_a_ and *D*_b_ (see Table 3). We also simulated smaller genetic effects to further challenge the method.
3. Set 3. The third simulation set tested the scenario in which there were no pleiotropic SNPs, to assess the “false discovery rate” of the method and the effect on prediction. Unlike the other two sets, the third simulation set consisted of only two scenarios, one with moderate effects (OR 1.25–2.0) and one with strong effects (OR 1.75–2.5). Each scenario was applied to 100 simulated GWAS. All GWAS contained 150 causal SNPs: 75 associated only with *D*_a_, and 75 associated only with *D*_b_. Other than these exceptions, these scenarios were identical to those in simulation set 1.
4. Set 4. The final simulation set tested the classifier performance in two scenarios with a wide variation in the effect strengths of the individual causal SNPs (Table 4). These two scenarios were run almost identically to the scenarios from simulation set 1, except in both the odds ratios were drawn from a distribution ranging from 1.1 to 2.5. The first scenario set simulated large 4000-subject discovery sets, the second scenario simulated smaller 1500-subject discovery sets.

**Table 1:** **Set 1 scenario parameters**.

No.	Scenario name	Sample size	OR_min_	OR_max_
1	1.5k Sample, Weak effect	4000	1.10	1.50
2	4k Sample, Weak effect	1500	1.10	1.50
3	1.5k Sample, Moderate effect	4000	1.25	2.00
4	4k Sample, Moderate effect	1500	1.25	2.00
5	1.5k Sample, Strong effect	4000	1.75	2.50
6	4k Sample, Strong effect	1500	1.75	2.50

**Table 2:** **Set 2 discovery and validation set sample size and phenotype distribution**.

	Discovery set		Total		Validation set		Total
	*D*_a_ = 1	*D*_a_ = 2			*D*_a_ = 1	*D*_a_ = 2	
***D*_b_ = 1**	450	50	500	***D*_b_ = 1**	1800	200	2000
***D*_b_ = 2**	450	50	500	***D*_b_ = 2**	1800	200	2000
**Total**	900	100	1000	**Total**	3600	400	4000

**Table 3:** **Set 2 scenario parameters**.

No.	Scenario name	*D*_a_		*D*_b_	
		OR_min_	OR_max_	OR_min_	OR_max_
1	Weak/Moderate	1.1	1.5	1.5	2.0
2	Weak/Strong	1.1	1.5	2.0	2.5
3	Moderate/Moderate	1.5	2.0	1.5	2.0
4	Moderate/Strong	1.5	2.0	2.0	2.5
5	Strong/Moderate	2.0	2.5	1.5	2.0
6	Strong/Strong	2.0	2.5	1.5	2.5

**Table 4:** **Set 4 scenario parameters**.

No.	Scenario name	Sample size	OR_min_	OR_max_
1	4k Sample	4000	1.1	2.5
2	1.5k Sample	1500	1.1	2.5

### Simulation set 1 results: Pleiotropy with balanced phenotypes

**Figure 1:**
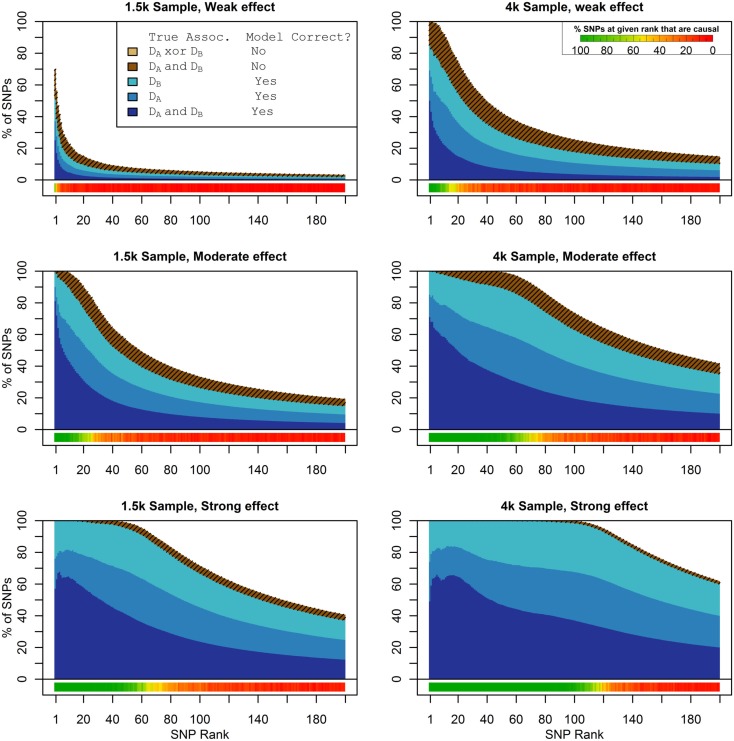
**Nested model composition, 2-phenotype search (Simulation Set 1)**. The six graphs depict, for each of the six scenarios, the composition of the models resulting from the 2-phenotype phase I model search (y-axis), as a function of SNP rank cutoff (x-axis). The three blue, un-shaded colors indicate the percentage of the SNPs in the given models that were both causal and assigned to the correct association models. The dark blue indicates pleiotropic SNPs, the medium blue indicates *D*_a_-associated SNPs, and the light blue indicates *D*_b_-associated SNPs. The brown colors (with diagonal shading lines) indicate SNPs that are causal, but were assigned the incorrect model. Dark brown indicates pleiotropic SNPs that were incorrectly assigned a single-phenotype model. Tan (with diagonal shading lines) would indicate single-phenotype-associated SNPs that were incorrectly assigned either the pleiotropic model or a model with the wrong SNP, but this happened so infrequently that no visible tan pixels are visible. The remaining white space indicates non-causal SNPs erroneously included in the nested models. For example, in the “4k sample, moderate effect” scenario (mid-right plot), the 80-SNP model contains approximately 30% pleiotropic SNPs, 25% SNPs associated with *D*_a_ and *D*_b_ each, and around 10% pleiotropic SNPs mistakenly assigned a single-SNP associated model, and around 10% non-causal SNPs (white space). Beneath each graph is a color bar summarizing the percentage of causal SNPs discovered at each rank (see key, inset).

**Figure 2:**
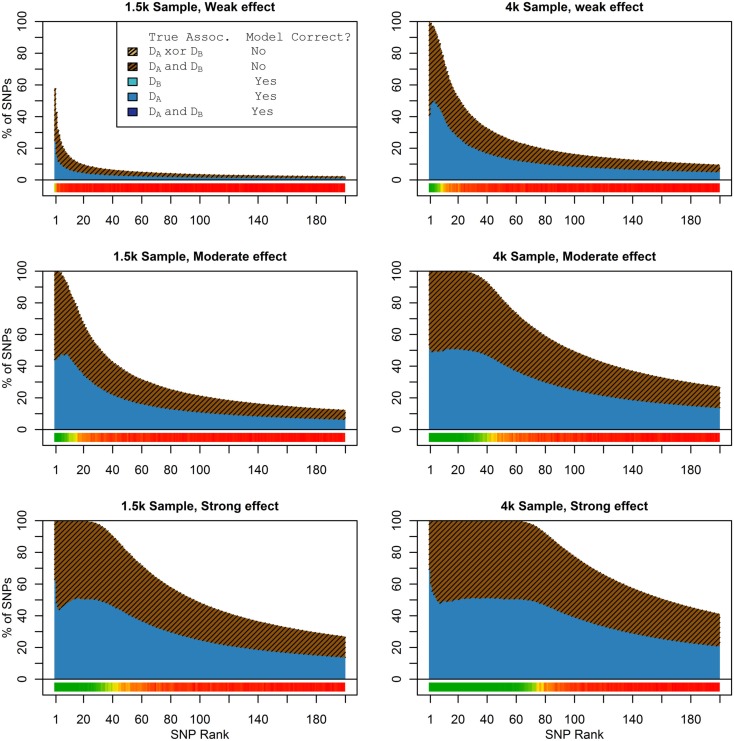
**Nested model composition, single-phenotype (naive) search (Simulation Set 1)**. As Figure 1, except for the single-phenotype (naive) model search, in which *D*_b_ is entirely withheld from the model selection process. Note that since no pleiotropic models were fitted, all pleiotropic SNPs that were discovered were of course incorrectly modeled with the single-phenotype model. Furthermore, all correctly modeled SNPs were always associated with *D*_a_ only.

Figure 1 shows the true discovery rate (across 100 simulations) of the pleiotropic model search described in the methods, while Figure 2 shows the true discovery rate for the naive model search in which one of the two phenotypes was ignored. The solid colors indicate the total percentage of the SNPs that were identified as causal and assigned the correct model, whereas the shaded colors indicate SNPs that were identified as causal but assigned an incorrect model. The *x*-axis indicates the ranking of the last SNP included in the nested SNP sets, i.e., the number of SNPs in the SNP sets. The color bar at the bottom of each graph indicates the percentage of SNPs at each specific ranking that is true causal across 100 simulations (e.g., the percentage of causal SNPs for the 50th SNP *S*_50_ across 100 simulations, as opposed to the percentage of causal SNPs in the SNP set Σ_50_ = {*S*_1_, …, *S*_50_} across 100 simulations).

As we would expect, larger samples and stronger effects result in higher true discovery rates. Furthermore, the pleiotropic SNPs are more often highly ranked when modeled fully using the 2-phenotype search than when only partially modeled as being single-phenotype associated (Figure 2). Thus, the pleiotropic search method not only models these SNPs correctly (as opposed to the naive search, which will only model them as single-phenotype-associated), but it also ranks them higher and thus has a higher true discovery rate.

**Figure 3:**
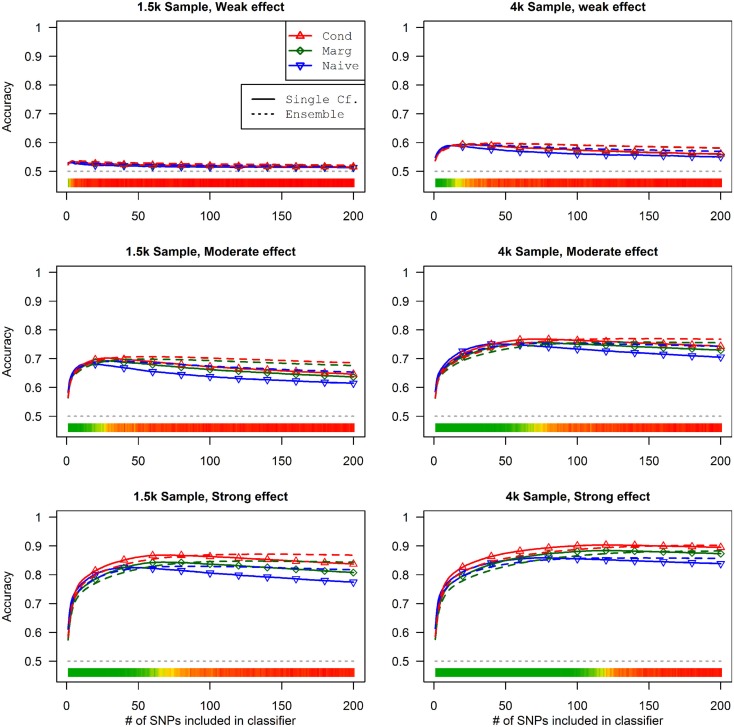
**Accuracy of single-classifier and ensemble prediction of *D*_a_ using three types of classification, by SNP rank cutoff (Simulation Set 1)**. For each of the six scenarios, the accuracy of single-classifier (solid lines) and ensemble-of-classifier (dashed lines) prediction of *D*_a_, using three prediction methods: conditional prediction, in which *D*_a_ is predicted given known *D*_b_ (red, upward triangles); marginal, in which *D*_a_ is predicted without known *D*_b_ (green, diamonds); and the single-phenotype prediction, in which model search and classification accounts only for *D*_a_ (blue, downward triangles).

This advantage translates into higher accuracy in the prediction (see Figure 3). In all scenarios, the conditional prediction of *D*_a_, given both genotype data and *D*_b_ status, has the highest peak accuracy, followed by the marginal prediction (in which predictions are made using the pleiotropic model but assuming *D*_b_ status is unknown), and, with the lowest peak accuracy, the naive prediction (in which both model search and prediction was carried out without using *D*_b_). For each prediction statistic type, the ensemble predictions achieved slightly higher peak accuracy than the single-classifier prediction. Additionally, the ensemble prediction generally peaked when using far more SNPs, including a large proportion of non-causal SNPs, and appears to be more robust to the inclusion of false positive SNPs.

Similarly, using the final cross-validation-selected classifiers, the conditional prediction achieved the highest accuracy, followed by the marginal prediction and the naive prediction. Accuracy was of course higher with larger sample sizes and stronger effects, and ranged between 53.4 and 90.3% for the conditional prediction, 53.3 and 88.3% for the marginal prediction, and 52.8 and 85.7% for the naive prediction (see Table 5). Note that, in the case of a balanced phenotype, the classification accuracy based on the Bayesian rule (threshold = 1) performs as well as an optimized classification rule chosen via cross-validation.

**Table 5:** **Phase II selected model prediction accuracy, by search and prediction method**.

No.	Classification rule threshold	(Specificity + sensitivity)/2 (mean ± SD)		
		Naive single Cf.	Marginal single Cf.	Conditional single Cf.
**SIMULATION SET 1**				
1	Threshold = 1	0.528 ± 0.03	0.533 ± 0.03	0.534 ± 0.03
	Cross-val thresh	0.529 ± 0.03	0.532 ± 0.03	0.532 ± 0.03
2	Threshold = 1	0.589 ± 0.02	0.592 ± 0.02	0.593 ± 0.03
	Cross-val thresh	0.587 ± 0.02	0.591 ± 0.02	0.591 ± 0.03
3	Threshold = 1	0.680 ± 0.03	0.690 ± 0.03	0.699 ± 0.04
	Cross-val thresh	0.678 ± 0.03	0.688 ± 0.03	0.697 ± 0.03
4	Threshold = 1	0.751 ± 0.02	0.755 ± 0.02	0.767 ± 0.02
	Cross-val thresh	0.750 ± 0.02	0.753 ± 0.02	0.766 ± 0.02
5	Threshold = 1	0.824 ± 0.03	0.842 ± 0.03	0.866 ± 0.03
	Cross-val thresh	0.823 ± 0.03	0.841 ± 0.03	0.865 ± 0.03
6	Threshold = 1	0.858 ± 0.02	0.884 ± 0.02	0.903 ± 0.02
	Cross-val thresh	0.857 ± 0.02	0.883 ± 0.02	0.903 ± 0.02
**SIMULATION SET 2**				
1	Threshold = 1	0.500 ± 0.01	0.504 ± 0.01	0.505 ± 0.02
	Cross-val thresh	0.501 ± 0.01	0.504 ± 0.02	0.504 ± 0.02
2	Threshold = 1	0.501 ± 0.01	0.505 ± 0.01	0.507 ± 0.02
	Cross-val thresh	0.499 ± 0.01	0.505 ± 0.01	0.507 ± 0.01
3	Threshold = 1	0.528 ± 0.03	0.579 ± 0.06	0.583 ± 0.06
	Cross-val thresh	0.539 ± 0.04	0.586 ± 0.06	0.593 ± 0.06
4	Threshold = 1	0.524 ± 0.03	0.595 ± 0.04	0.608 ± 0.05
	Cross-val thresh	0.529 ± 0.04	0.593 ± 0.04	0.608 ± 0.05
5	Threshold = 1	0.694 ± 0.08	0.746 ± 0.06	0.754 ± 0.07
	Cross-val thresh	0.707 ± 0.07	0.748 ± 0.06	0.758 ± 0.06
6	Threshold = 1	0.557 ± 0.05	0.652 ± 0.05	0.684 ± 0.05
	Cross-val thresh	0.570 ± 0.05	0.652 ± 0.05	0.686 ± 0.05
**SIMULATION SET 3**				
1	Threshold = 1	0.616 ± 0.05	0.618 ± 0.05	0.618 ± 0.04
	Cross-val thresh	0.615 ± 0.05	0.616 ± 0.05	0.616 ± 0.05
2	Threshold = 1	0.783 ± 0.04	0.781 ± 0.04	0.780 ± 0.04
	Cross-val thresh	0.781 ± 0.04	0.780 ± 0.04	0.779 ± 0.04
**SIMULATION SET 4**				
1	Threshold = 1	0.817 ± 0.03	0.826 ± 0.03	0.841 ± 0.03
	Cross-val thresh	0.816 ± 0.03	0.825 ± 0.03	0.840 ± 0.03
2	Threshold = 1	0.793 ± 0.03	0.801 ± 0.03	0.816 ± 0.03
	Cross-val thresh	0.792 ± 0.03	0.800 ± 0.03	0.814 ± 0.03

*Reports, for the three simulation sets, and for each of the single-classifier classification rules, the mean and standard deviation of the average of the specificity and sensitivity for the classification of the external replication sets using the final models selected by the cross-validation algorithm. Both the Bayesian 0–1 loss function threshold (i.e., threshold = 1) and the cross-validation selected threshold (in which the threshold is selected so as to maximize Youden’s *J* in the cross-validation) are listed. The conditional and marginal classifications are based on the pleiotropic search method; the conditional classification uses both the subject genotype and the secondary phenotype status, whereas the marginal classification is based only on the subject genotype. The naive classification uses the naive search method to find the model, and as such does not use the secondary phenotype in either model selection or classification*.

### Simulation set 2 results: Pleiotropy with unbalanced phenotypes

**Figure 4:**
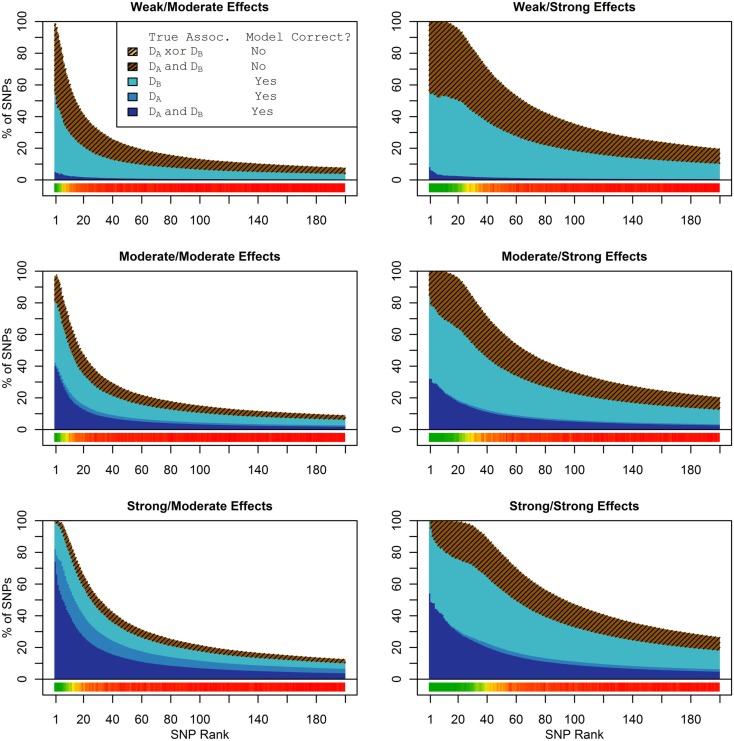
**Set 2  Total model composition by rank, 2-phenotype model search (Simulation Set 2)**. See legend for Figure 1.

**Figure 5:**
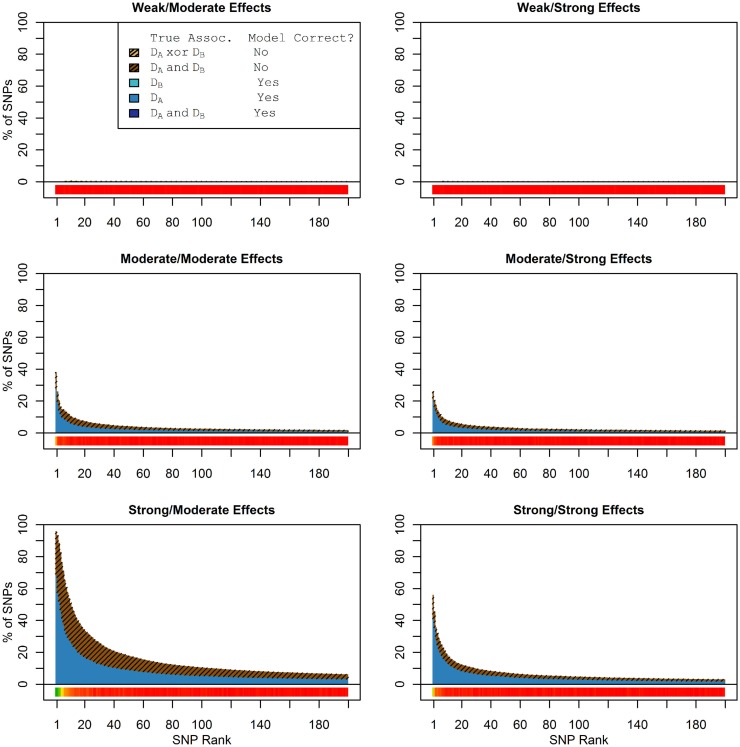
**Nested model composition by rank, single-phenotype model search (Simulation Set 2)**. See legend for Figures 1 and 2.

In these simulations, the pleiotropic model search had an even larger advantage compared to the naive search, than it did in the simulation set 1. With an unbalanced case-control ratio, the power for detecting associations with *D*_a_ was substantially weaker than the power for detecting associations with *D*_b_, even when the effect strengths were the same. As a result, relatively few SNPs associated only with *D*_a_ were ranked highly in the pleiotropic search (see Figure 4), and very few of either type of *D*_a_-associated SNPs (pleiotropic or *D*_a_ only) were ranked highly by the naive search (see Figure 5).

**Figure 6:**
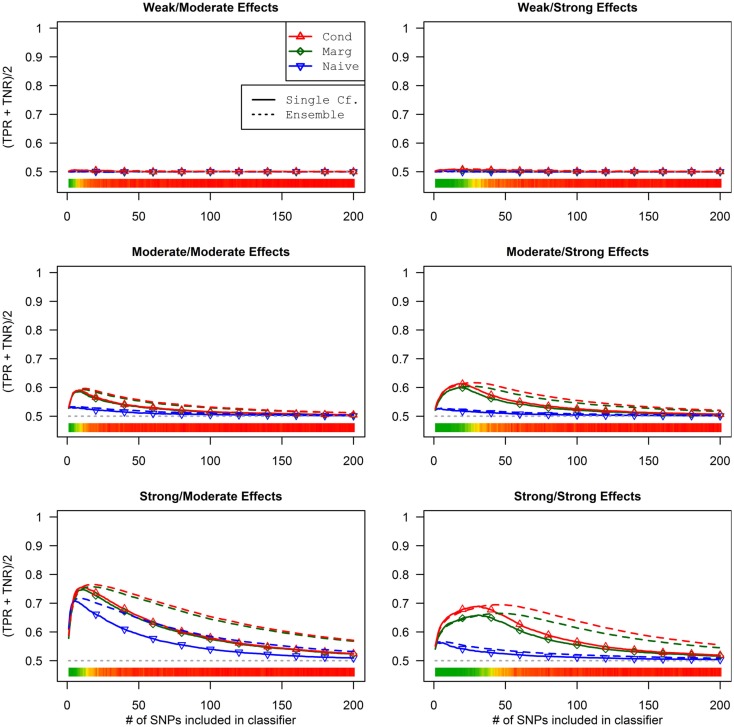
**Single-classifier and ensemble-of-classifier *D*_a_ prediction using three prediction methods, with both single-classifier and ensembles, by SNP set size (Simulation Set 2)**. See legend for Figure 3. Due to the uneven distribution of *D*_a_, total accuracy is not a useful measure of prediction. Therefore, the average of the true positive rate (sensitivity) and true negative rate (specificity) was used. Note that this is a simple linear transformation of the Youden’s *J* statistic, used to make it comparable to the simple accuracy statistic used in simulation set 2.

The conditional and marginal prediction performed much better than the naive prediction, although the accuracy for all methods was lower than in the first set of simulations (see Figure 6). The difference between the pleiotropic and the naive methods was much larger than seen in the first set of simulations. As before, the conditional prediction was slightly better than the marginal prediction, for both ensemble and single-classifier classification rules. Similarly, the ensemble prediction rules had slightly higher peak accuracy than the single-classifier rules, and peaked with much larger final SNP sets.

It is worth noting that unlike in simulation set 1, the threshold selection provided a considerable improvement in the prediction relative to the Bayesian classification rule (see Table 5).

### Simulation set 3 results: No pleiotropy

**Figure 7:**
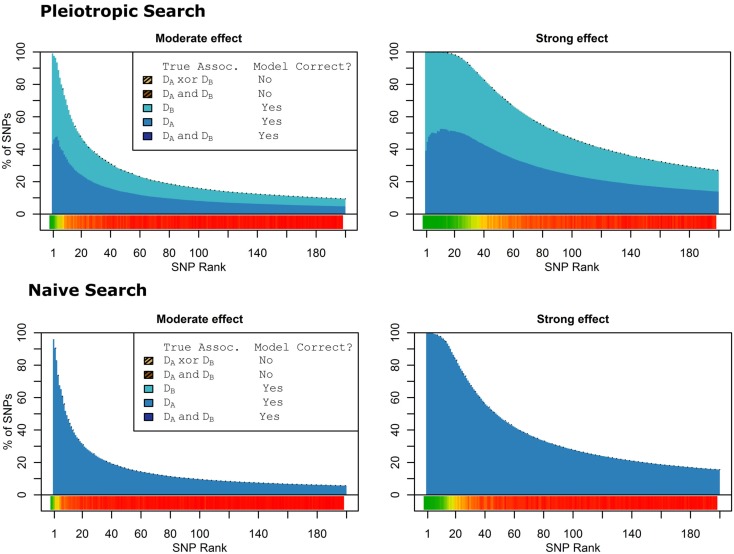
**Nested model composition by SNP set, pleiotropic, and naive model searches (Simulation Set 3)**. Legend as in Figures 1 and 2.

When there were no true pleiotropic SNPs in either of these scenarios, neither model composition plot shows any dark blue. The only substantive difference between the naive and pleiotropic model search algorithms was that the pleiotropic search modeled some single-phenotype-associated SNPs as being associated with both phenotypes, but this false discovery rate was small (see Figure 7). In fact, in Figure 7, only a few pixels are visible that corresponded to SNPs that were mistakenly applied to the pleiotropic model. Additionally, very few of the non-causal SNPs were falsely discovered as being pleiotropic (see Table 6 for an example).

**Table 6:** **Model assignment by SNP type, for each search method (using BF threshold: ln(BF) > 1) (Simulation Set 3)**.

2-Phenotype search				Naive search			
Assigned model	True association			Assigned model	True association		
	*D*_a_ only	*D*_b_ only	Non-causal		*D*_a_ only	*D*_b_ only	Non-causal
**MODERATE EFFECT SCENARIO**							
*D*_a_ and *D*_b_	0.0005	0.0023	0.0002	*D*_a_ and *D*_b_	0	0	0
*D*_a_ only	**0.4181**	0.0185	0.0267	*D*_a_ only	**0.4205**	0.0271	0.0272
*D*_b_ only	0.0196	**0.4168**	0.0266	*D*_b_ only	0	**0**	0
Non-causal	0.5617	0.5624	**0.9464**	Non-causal	0.5795	0.9729	**0.9728**
**STRONG EFFECT SCENARIO**							
*D*_a_ and *D*_b_	0.0016	0.0024	0.0002	*D*_a_ and *D*_b_	0	0	0
*D*_a_ only	**0.7077**	0.0107	0.0267	*D*_a_ only	**0.7124**	0.0285	0.0272
*D*_b_ only	0.0093	**0.6957**	0.0266	*D*_b_ only	0	**0**	0
Non-causal	0.2813	0.2912	**0.9464**	Non-causal	0.2876	0.9715	**0.9728**

*Displays the rate at which each SNP type was assigned each model, for both the naive and 2-phenotype model searches. Recall that all SNPs that did not pass the first-pass significance threshold of ln(BF) > 1 were assigned the null model, and otherwise were assigned the most likely of the three models. The rates at which each SNP type were assigned the correct respective models are listed in bold*.

**Figure 8:**
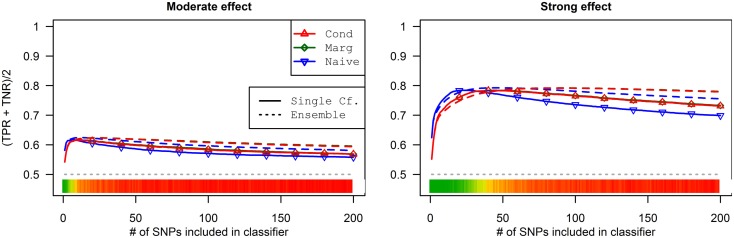
**Accuracy by prediction method and number of SNPs used, by prediction method (Simulation Set 3)**. Legend as in Figure 3.

As a result, the prediction accuracy was about the same across all prediction statistics. The only real difference between the naive prediction and the pleiotropic prediction was that the pleiotropic prediction accuracy peaked with about double the number of SNPs, as it included SNPs associated with both *D*_a_ and *D*_b_. Since there were very few SNPs modeled as pleiotropic, the marginal and conditional prediction statistics were almost identical, and both were almost identical to the corresponding naive prediction statistic (see Figure 8; Table 7). The cross-validation similarly found almost-equivalent classifiers for all three types of classification statistics (see Table 5).

**Table 7:** **Peak accuracy by prediction method (Simulation Set 3)**.

Prediction method		Moderate effect		Strong effect	
		Peak Acc.	No. of SNPs	Peak Acc.	No. of SNPs
Naïve	S. Cf.	0.618635	7	0.784248	22
	Ens.	0.624560	11	0.792385	45
Marginal	S. Cf.	0.616150	12	0.784140	44
	Ens.	0.623878	24	0.792025	93
Conditional	S. Cf.	0.615453	12	0.783708	44
	Ens.	0.623358	24	0.791498	90

### Simulation set 4: Pleiotropy with wide variation in effect strength

**Figure 9:**
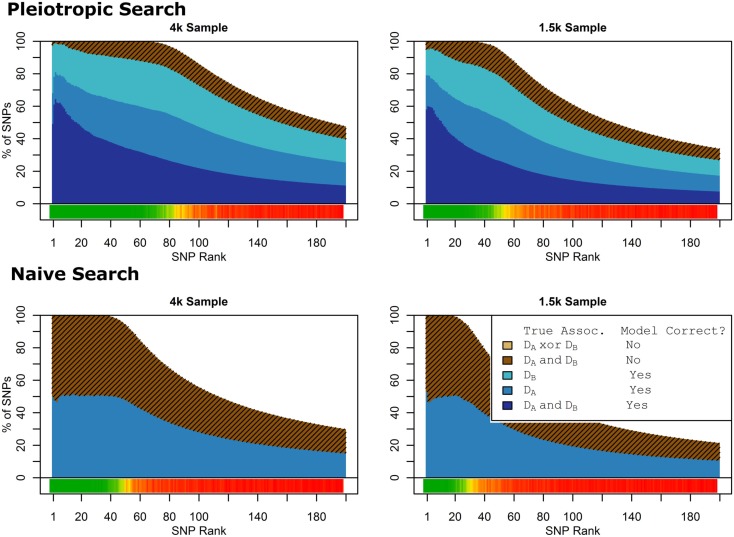
**Model composition by SNP set, pleiotropic, and naive model searches (Simulation Set 4)**. Legend as in Figures 1 and 2.

**Figure 10:**
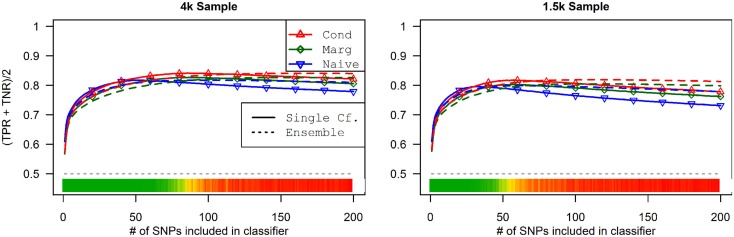
**External validation AUC of the ROC curve, with 95% CI (Simulation Set 4)**. Legend as in Figure 3.

As seen in Figures 9 and 10, the model search and classification does not suffer any new problems when the causal SNPs vary widely in effect strength. The analyses worked about as expected: better than the moderate effect-strength scenarios from simulation set 1, but worse than the strong effect scenarios. As before, the conditional prediction performed best (up to 0.841 accuracy), followed by the marginal prediction (up to 0.825 accuracy), and the naive prediction (up to 0.817 accuracy), see Table 5.

### Results of testing with real data

Data from five different studies of sickle cell disease (SCD) patients were collected and used to test the proposed methods under real world conditions. The primary phenotype of interest was cerebral vascular accident (CVA), or stroke, a complication seen in about 10% of children with SCD. The secondary phenotype was fetal hemoglobin level (HbF), a laboratory measurement that has previously been found to be associated with reduced mortality and improved clinical prognosis.

The first and largest of the five studies, the Cooperative Study of SCD (CSSCD), was used as the discovery set. A total of 1071 subjects were taken from the CSSCD, 83 of which had reported CVA.

Four smaller study datasets were used for validation: the Multicenter Study of Hydroxyurea (MSH), Pulmonary Hypertension and the Hypoxic Response in SCD (PUSH), Treatment of Pulmonary Hypertension and SCD with Sildenafil Treatment (walk-PHaSST, or WP), and the Comprehensive Sickle Cell Centers Collaborative Data Project (C-Data). In total, the validation dataset consisted of 352 subjects, 37 of which had reported CVA.

**Figure 11:**
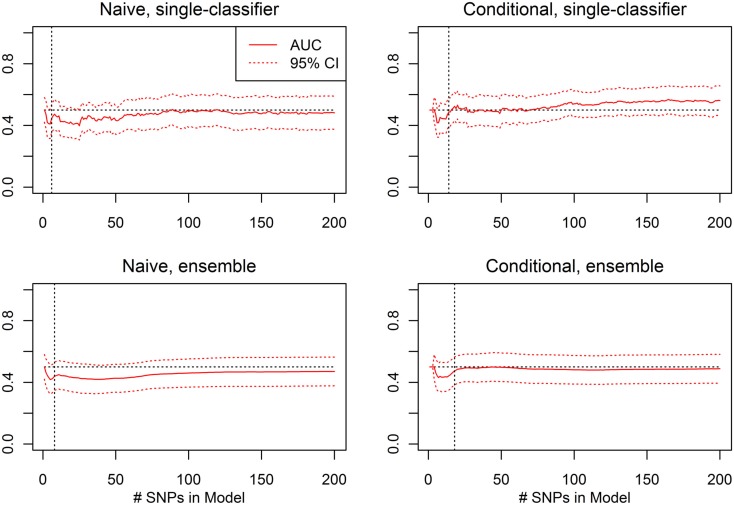
**External validation AUC of the ROC curve, with 95% CI (Real Data Set)**. Validation set AUC, with Delong 95% confidence intervals, using the classification statistics calculated from each of the nested SNP sets Σ_1_, …, Σ*_r_*, for each of four prediction methods: naive single-classifier, naive ensemble, conditional single-classifier, and conditional ensemble.

For each prediction method (conditional single-classifier, conditional ensemble, naive single-classifier, and naive ensemble), and each nested model Σ_1_, …, Σ_200_, leave-one-out cross-validation was used to select an optimal decision threshold for prediction of CVA given known HbF. These classifiers were tested on the external validation set for which HbF data was available, which was comprised of 352 subjects, 37 of which were CVA cases. Any differential classification was generally not even nominally statistically significant, and the few nested models that did yield single-test statistical significance would not remain significant after correction for multiple testing (see Figure 11).

The average of the sensitivity and the specificity for the single-classifier naive prediction peaked in the validation set at 0.530 (using classifier Σ_151_, with 151 SNPs), and for the ensemble naive prediction peaked at 0.501 (using an ensemble of classifiers based on nested models Σ_1_ to Σ_2_). The average of sensitivity and specificity for the conditional single-classifier was generally slightly higher than 0.5, and peaked at 0.601 (using classifier Σ_97_ with 97 SNPs). The conditional ensemble classifier peaked at 0.549 (using an ensemble of classifiers based on nested models Σ_1_ to Σ_23_). Both naive prediction methods yielded approximately random prediction areas under the receiver operating curve (AUC) (see Figure 11). The AUCs were similar, and although none of the four methods achieved statistical significance, the conditional prediction, which used information on HbF status as well as genotype, performed slightly (but not significantly) better than the naive prediction (Figure 11). The lack of statistical significance is likely due to the extremely small number of cases in the validation set (only 37 subjects had a CVA event). Note also that in this analysis, the cross-validation-selected SNP sets did not perform as well as the peak classifiers. All four classification methods performed about as would be expected by random chance (see Table 8).

**Table 8:** **Prediction summary statistics of cross-validation-selected classifiers, by method (Real Data Set)**.

	Naive prediction				Conditional prediction			
	Single Cf.		Ensemble		Single Cf.		Ensemble	
No of SNPs	6		8		14		18	
Prediction thresh	0.561825199		0.606951538		0.508542101		0.622242802	
Sensitivity	0.189189189		0.432432432		0.108108108		0.513513514	
Specificity	0.685714286		0.514285714		0.780952381		0.492063492	
Mean (sens, spec)	0.437451737		0.473359073		0.444530245		0.502788503	
AUC	0.445945946		0.43963964		0.4874		0.474646075	
AUC 95% CI	0.3570	0.5349	0.3490	0.5303	0.3958	0.5790	0.3841	0.5652

*Various summary statistics for prediction using the final SNP sets selected via the cross-validation algorithm. The AUC 95% confidence intervals are calculated using the Delong method described in Delong et al. (McKinney et al., 2006), implemented via the pROC package in R*.

## Discussion

We presented a new method to discover SNPs that are associated with multiple traits and a model based approach to risk prediction that uses pleiotropic SNPs to increase accuracy. We evaluated the proposed approach in four simulation sets. In simulation set 1, we demonstrated that the methods function well on large datasets with strong genetic effects. Many pleiotropic loci were detected by the model search methods, and very few SNPs were misidentified as being pleiotropic. Furthermore, the pleiotropy-based prediction methods showed a substantial improvement over standard naive classification, particularly when the value of the secondary phenotype was known. In the second simulation set, we demonstrated that these methods also function well when applied to an unbalanced case-control dataset, and in fact the improvement over the naive methods was even more substantial in these scenarios. In the third simulation set, we demonstrated that the methods do not perform significantly worse than conventional analysis when applied to data in which pleiotropy is absent. In the final simulation set, we showed that with genetic effects of varying strength, the methods perform as expected. Very few SNPs were falsely assigned the pleiotropic model by the model search, and as such the prediction by all three methods were very similar. The simulation results are consistent with the hypothesis that genetic data can help prediction when the effects are large, and the predictive accuracy increases with larger sample sizes. Although we did not investigate the specific effects of minor allele frequencies, we expect that large samples will be needed to accurately estimate the genetic effects of rare variants.

Although there was no statistically significant differential prediction in the CVA/HbF analyses, we did demonstrate that these methods can be applied to real data, and that the conditional prediction may perform better than the naive prediction in certain circumstances. It should be noted that given the small validation set sample size and the relatively low heritability of CVA, the lack of significant validation is not surprising.

As genome-wide assays have rapidly become more affordable, genome-wide data has become correspondingly more commonplace. Correction for multiple comparisons often results in low power, particularly for weak, multigenic associations. Assuming pleiotropic associations are indeed present within a dataset, these methods may be able to achieve higher power than analyses using the same data that only search for single-phenotype associations. Additionally, by leveraging pleiotropy, these methods may be able to more accurately predict phenotype status than traditional naive Bayesian classifiers. The model search and classification methods described in this paper are capable of effective pleiotropic locus identification and phenotype classification under a variety of conditions.

The approach described uses simple Bayesian networks for classification, built by essentially merging and retraining simple Naive Bayes classifiers. One of the problems of naive Bayes classifiers is determining the optimal number of features. Including too many SNPs can reduce prediction accuracy due to the large proportion of false positive associations included in the model, while applying stringent genome-wide significance thresholds can yield sub-optimal prediction, as the reduction in false positives comes at the cost of a reduction in true positives. However, particularly with weak genetic effects, there may be valid multiple-comparison concerns with classifier validation: if there is found to be only a narrow range of SNP set sizes within which the classifier predicts significantly better than chance, then that narrow range could potentially be dismissed as the result of random chance. Therefore, the cross-validation methods described here represent an attempt at finding a single final SNP set that can be expected to yield reasonable (if not optimal) prediction. Their application to both simulated and real data show mixed results, and further refinement of these algorithms may yet be necessary.

An alternative approach to search for the “best classifier” is to use an ensemble of classifiers, and our results show that this approach can be more robust to inclusion of false positive associations. Ensemble of classifiers is well known to improve prediction and many methods have been proposed (Rokach, 2010). The approach investigated here can be improved using more advanced ensemble methods.

An additional limitation of Naïve Bayes classifiers is the assumption that SNPs are conditionally independent given the phenotype. We have recently shown that this assumption makes the prediction rule based on a Naïve Bayes classifier equivalent to the more popular approach of collapsing genetic information into a genetic risk score but it provides a more general modeling framework that leads, for example, to the pleiotropic modeling introduced in this manuscript (Sebastiani et al., 2012b). However, the impact of more complex dependency structure among SNPs on the predictive accuracy needs to be investigated further. Bayesian network models would provide an ideal extension of this approach to include more general dependency structures between SNPs (Jiang et al., 2011).

Finally, the Bayesian model approach lends itself naturally to genetic risk prediction, and the more accurate modeling of phenotype-genotype associations used by these methods can provide improved prediction over analogous single-phenotype prediction methods, even absent additional information from the subjects being predicted (i.e., using the marginal classification).

In summary: our proposed methods have two distinct applications. First, model selection and subsequent replication set prediction can be used to identify and verify potential candidate genes for additional study. Particularly in the case of multigenic diseases that are governed by numerous weakly penetrant causal variants, genome-wide significance levels may be far too conservative, and direct replication may provide similarly inadequate power. By combining many weak effects, genetic risk prediction can be used to validate the composite of numerous causal loci. Secondly, these classification scores could be directly applied to develop novel diagnostic and prognostic tests.

The methods proposed here are highly extensible, and can easily be expanded to account for more than two phenotypes, correlated phenotypes, and/or additional covariates and phenotypes. Phenotypes that are marginally correlated can be easily included in the model specification and will only change the formulation of the predictive probabilities described in Section “Model Search, Phase I: Discovery of Significant SNPs and Generation of Nested Models.” The correct formulation can be derived from the specific assumptions of conditional independence, using for example algorithms derived for reasoning with Bayesian networks (Shriner, 2012). We expect that the impact of correlated phenotypes on the predictive accuracy will depend on the strength of the correlation between phenotypes and future studies will elucidate this further. Additional covariates can be easily included if they are qualitative variables, while inclusion of continuous covariates would probably require Markov Chain Monte Carlo methods to estimate the predictive probability of phenotypes given genetic data and a set of covariates. Additional phenotypes would increase the degrees of freedom, and such extensions would require significantly larger samples; however, as genotype assays grow more affordable, sufficiently large datasets may become more common. Loci associated with more than two phenotypes would be even more likely to be central in the Human PPI network, and thus might be more likely to possess essential functional significance. Furthermore, as these associations are predicated on this complex network of underlying protein interactions, building classification scores that condition on multiple known phenotypes may improve prediction even further.

With the rapid expansion and proliferation of genetic, expression, phenome, and protein–protein interaction datasets, new methods must be developed to efficiently extract useful meaning from the overwhelmingly complex network of (unknown) underlying biological mechanisms. The usage of pleiotropy and genetic risk prediction to improve candidate SNP identification and to develop novel prognostic tests represent just one of many approaches aimed at leveraging these interactions toward the extraction of practical information.

## Methods

The algorithm takes as input a genome-wide association study dataset with multiple known and potentially related phenotypes, identifies relationships between the SNPs and the phenotypes, and uses these relationships to generate classifiers and ensembles of classifiers that can predict one or multiple target phenotypes.

The algorithm operates in two distinct phases: in phase I, the SNPs are ranked by significance of association, and the most likely association model is determined for each SNP. This yields a series of ranked SNPs, which can be used to build nested Bayesian classification rules by adding one SNP at a time from the ranked list. In phase II, the optimal number of SNPs is estimated via 10-fold cross-validation.

Although these methods can easily be extended for use with three or more phenotypes, we will limit our focus to the investigation of two dichotomous phenotypes: *D*_a_ and *D*_b_, each taking values of 1 or 2.

### Overview of bayesian modeling framework

Let *S* be a random variable representing a single-SNP, with 2–3 possible values, depending on the mode of inheritance being tested. In the recessive mode, *S* is modeled as a Bernoulli random variable with two possible values: 1 = {AA | AB} and 2 = {BB}. In the dominant mode, *S* is coded as 1 = {AA} and 2 = {AB | BB}. In the allelic mode, each allele is treated as a separate observation, with 1 = {A} and 2 = {B}. Finally, in the genotypic model, *S* is modeled as a categorical random variable with three possible values: 1 = {AA}, 2 = {AB}, and 3 = {BB}.

We did not include the additive model, as it is very similar to the allelic model.

We model the SNP random variables as having distributions that are conditional on the phenotype class, and then the classification rules are computed by using Bayes theorem to calculate the probability of the phenotype given a genetic profile (Sebastiani et al., 2012a).

For each SNP *S*, we consider four possible relationships between *D*_a_, *D*_b_, and *S*: *M*_0_, the null model, in which the distribution of the SNP is independent of either phenotype; the single-phenotype association models *M*_a_ and *M*_b_, in which the genotype frequencies of *S* is dependent on *D*_a_ or *D*_b_, respectively; and *M*_ab_, the pleiotropic model, in which the distribution of *S* is dependent on both *D*_a_ and *D*_b_. We assume that these four models are, *a priori*, equally likely.

The model selection process has three major goals: (1) determine which of these models is the most likely, (2) measure the strength of the evidence for the associations, and (3) use this information to produce effective risk prediction for the traits *D*_a_ and *D*_b_.

### Model search, phase I: Discovery of significant SNPs and generation of nested models

In phase I, the most likely model for each SNP is calculated using a Bayesian method, and the strength of the evidence of this association is measured. The SNPs are then ranked in descending order of the posterior odds of association. Since uniform prior probabilities are used, the posterior odds are equivalent to Bayes factors, defined below (Balding, 2006).

First, for each SNP *S*, single-phenotype Bayes factors are calculated for each phenotype *D*_a_ and *D*_b_. These Bayes factors compare the likelihood of observed genotypes $\vec{S}$ given observed phenotypes ${\vec{D}}_{\text{a}}$ and ${\vec{D}}_{\text{b}},$ under the models in which the distribution of the SNP depends on one and only one of the two phenotypes (*M*_a_ or *M*_b_), with the likelihood of $\vec{S}$ under the null model (*M*_0_) in which the distribution of the SNP is independent of all phenotypes:

$$\text{B}{\text{F}}_{\text{a}\;\text{vs}\text{.}\;0}=\frac{p\left[\vec{S}|{\vec{D}}_{\text{a}},M_{\text{a}}\right]}{p\left[\vec{S}|M_{0}\right]}\;\text{and}\;\text{B}{\text{F}}_{\text{b}\;\text{vs}\text{.}\;0}=\frac{p\left[\vec{S}|{\vec{D}}_{\text{b}},M_{\text{b}}\right]}{p\left[\vec{S}|M_{0}\right]}$$

These calculations are carried out under the four different modes of inheritance: genotypic (2df), allelic, dominant, and recessive (see reference Sebastiani et al., 2012a for details), so that eight models are tested against the null hypothesis of no association.

Of the eight models tested in the first-pass, the model with the largest Bayes factor is selected for each SNP. We then only consider SNPs whose Bayes factor satisfies a first-pass significance threshold of ln(BF) > 1. Let *t* equal the number of SNPs selected.

Next, the pleiotropic model is tested for each of the *t* remaining SNPs. If *D*_x_ is the phenotype chosen in the first-pass, then if:

$$p\left[\vec{S}|M_{\text{ab}},{\vec{D}}_{a},{\vec{D}}_{b}\right]>p\left[\vec{S}|M_{\text{x}},{\vec{D}}_{x}\right]$$

then the model *M*_{a,b}_ would be selected for this SNP. Otherwise, the first-pass model (either *M*_a_ or *M*_b_, whichever has the higher Bayes factor) would be selected.

Next, the SNPs are ranked based on the Bayes factor comparing their respective selected models against the corresponding null models. Let *S*_1_, …, *S_r_*, …, *S_t_* be the *t* SNPs that pass the first-pass significance threshold, ranked in order of descending Bayes factor.

We then define *t* nested SNP sets: Σ_1_, …, Σ*_r_*, …, Σ*_t_*, for all 0 < *r *≤ *t* as:

$$\underset{r}{\sum }=\left\{S_{1},\ldots ,S_{r}\right\}$$

Additionally, to serve as a basis for comparison, a second model search is performed, in which SNPs are also ranked using *D*_a_ only, without accounting for *D*_b_ at all. The method used is similar to the one described above; except that only one phenotype is used and thus pleiotropic models are not tested. This alternate method is more explicitly described elsewhere, and produces standard naive Bayes classifiers (Sebastiani et al., 2012a).

### Prediction

Three distinct forms of prediction can be tested, differing by the information provided on the discovery set to the model search algorithm and by the information provided on the subjects whose phenotypes are being predicted. For each type, prediction can be performed by either a single-classifier or an ensemble of classifiers.

The first two prediction methods are based on the use of Bayes theorem to calculate the probability of a set of phenotypes given a genotype. Let Σ*_r_* be the set of SNPs selected by the model search, and let $M_{1}^{*},\;\ldots ,\;M_{i}^{*},\;\ldots ,\;M_{r}^{*}$ be the selected models for each SNP *S*_1_, …, *S_i_*, …, *S_r_*. For the purposes of prediction we always used the genotypic model that is more general and includes all other models of inheritance. Then the probability of having phenotypes $\vec{d}=\left(d_{a},d_{b}\right)$ given genotypes $\vec{S}=\left(S_{1},\;\ldots ,\;S_{r}\right)$ is:

$$p\left[\vec{D}=\left(d_{a},d_{b}\right)|\vec{s},\underset{r}{\sum }\right]\propto P\left[\vec{D}=\vec{d}\right]\overset{r}{\underset{i=1}{\prod }}P\left[S_{i}=s_{i}\left|\right.\vec{D}=\vec{d},M_{i}^{*}\right]$$

which can be calculated using the Bayesian estimate of the conditional probability of the genotype given the phenotype for each SNP *S_i_*:

$$P\left[S_{i}=s_{i}|\vec{D}=\vec{d},M_{i}^{*}\right]=\frac{n_{ijs}+a_{v}}{n_{ij∙}+3\cdot a_{v}}$$

In the formula we define:

$$a_{v}=\frac{4}{q}$$

*n_ijs_* is the (*j*, *s*) cell in the contingency table of phenotype values vs. SNP values under model $M_{i}^{*}$ and SNP *S_i_*, *j* is the index for the row in that table that corresponds to the phenotype values $\vec{d}$ and *q* is the total number of rows in that table (i.e., the number of possible phenotype combinations for the phenotypes that are modeled as associated with *S_i_* under model $M_{i}^{*}$).

We then define the classification statistics:

(1) Marginal prediction: A prediction for only one of the phenotypes, *D*_a_, is desired, using only the subject genotype $\vec{s}$. The other phenotype is assumed unknown for prediction, but the classification rule is trained on a discovery set that includes both phenotypes.
  Single-classifier prediction statistic:
  $$\begin{array}{llll} C_{1}^{\left(\text{margSC}\right)}\left(\vec{S},r\right) & =\frac{p\left[D_{\text{a}}=2|\underset{r}{\sum }\right]}{p\left[D_{\text{a}}=1|\underset{r}{\sum }\right]} & & \\ & =\frac{\overset{2}{\underset{d_{b}=1}{\sum }}p\left[D_{\text{a}}=2,d_{b}|\underset{r}{\sum }\right]}{\overset{2}{\underset{d_{b}=1}{\sum }}p\left[D_{\text{a}}=1,d_{b}|\underset{r}{\sum }\right]} & & \end{array}$$
  Ensemble of classifiers prediction:
  $$C_{1}^{\left(\text{m}\arg\text{Ens}\right)}\left(\vec{S},r\right)=\frac{\frac{1}{r}\overset{r}{\underset{k=1}{\sum }}p\left[D_{\text{a}}=2|\underset{k}{\sum }\right]}{\frac{1}{r}\overset{r}{\underset{k=1}{\sum }}p\left[D_{\text{a}}=1|\underset{k}{\sum }\right]}$$
(2) Conditional prediction: As above, a prediction for only one of the phenotypes, *D*_a_, is desired, but now we assume that both the subject genotype and the value of *D*_b_ is known. Once again, the classification rule is trained using both phenotypes in the discovery set.
  Single-classifier prediction:
  $$\begin{array}{rcll} C_{1}^{\left(\text{condSC}\right)}\left(\vec{S},r,d_{b}\right) & =\frac{p\left[D_{\text{a}}=2|D_{\text{b}}=d_{b},\vec{s},\underset{r}{\sum }\right]}{p\left[D_{\text{a}}=1|D_{\text{b}}=d_{b},\vec{s},\underset{r}{\sum }\right]} & & \\ & =\frac{p\left[D_{\text{a}}=2,D_{\text{b}}=d_{b}|\vec{s},\underset{r}{\sum }\right]}{p\left[D_{\text{a}}=1,D_{\text{b}}=d_{b}|\vec{s},\underset{r}{\sum }\right]} & & \end{array}$$
  Ensemble-of-classifiers prediction:
  $$C_{1}^{\left(\text{condEns}\right)}\left(\vec{S},r,d_{b}\right)=\frac{\frac{1}{r}\overset{r}{\underset{k=1}{\sum }}p\left[D_{\text{a}}=2|D_{\text{b}}=d_{b},\vec{s},\underset{k}{\sum }\right]}{\frac{1}{r}\overset{r}{\underset{k=1}{\sum }}p\left[D_{\text{a}}=1|D_{\text{b}}=d_{b},\vec{s},\underset{k}{\sum }\right]}$$
(3) Naive prediction: To serve as a basis for comparison, these classification rules were compared to those based on naive Bayesian classifiers. In this case, the classification rule was trained using *D*_a_ alone, ignoring all data on *D*_b_ in the discovery set. Nested models were built composed only of *D*_a_-associated single-SNP models. Bayesian classification rules were built using these nested models, using methods similar to those described here, except that only a single-phenotype was used. Both single-classifier prediction and ensemble-of-classifiers prediction was carried out using these naive Bayesian classifiers. These alternative methods are described in more detail elsewhere (Sebastiani et al., 2012a).

For any of these six prediction statistics, two different classification rules are tested.

$${\hat{D}}_{\text{a}}=\{\begin{array}{c} 2\text{ if }C_{1}>T\\ \text{1 otherwise} \end{array}$$

First, the threshold *T *= 1 was used, which is the optimal classification rule assuming balanced priors and a 0–1 loss function (Hand, 2009). This is known as “the Bayesian classification rule.” Second, we calculated an alternate classification threshold by selecting the prediction statistic threshold that maximized the Youden’s *J* statistic (*J* = sensitivity + specificity − 1), which is the threshold recommended by Perkins and Schisterman for optimizing dichotomous prediction (Perkins and Schisterman, 2006).

### Model search, phase II: Discovery of the optimal number of SNPs

In phase I, *t* nested SNP sets Σ_1_, …, Σ*_r_*, …, Σ*_t_* are created. In phase II, the optimal number of SNPs to be used is determined via cross-validation. Either 10-fold or leave-one-out cross-validation (LOOCV) can be used.

First, the discovery dataset is split into cross-validation training/test sets. For each training/test set, phase I model selection is repeated on the training set, and the corresponding test set is classified using the resultant nested SNP sets Σ_1_, …, Σ*_t_*. For each of the four prediction statistics both the *T *= 1 prediction threshold and the cross-validation-selected prediction thresholds are tested. Finally the specificity and sensitivity of the each model is calculated at each model size *r* for each prediction method.

The final number of SNPs to include in the model is determined by finding the number of SNPs that, in the cross-validation achieves the highest area under the Receiver Operating Characteristic (ROC) curve. To find this threshold we used the pROC package in R (Robin et al., 2011).

### Implementation

The vast majority of analyses were carried out using a custom-built utility, which is intended for eventual public release. The utility can read genotype and phenotype data in the standard PLINK binary file format. It can carry out both naive analyses, as well as pleiotropic analyses on two or more phenotypes using a variety of different search algorithms, some of which are not documented in this paper. The data output is designed to be easily read by most statistical packages, and several companion R scripts have been developed to provide secondary analyses, and visualization, including AUC calculations and threshold selection using the pROC library. The utility is written primarily in Java, and uses Java Standard Edition v1.6.0 and R v2.14.

Using this implementation, these analyses can be carried out very quickly. On the simulation set I scenario 6 (4000 discovery set subjects, 4000 replication set subjects, 500,000 SNPs each), running on a workstation with four Intel Xeon 2.4 GHz, quad-core processors and 64 Gb of RAM, the data read and phase I model search and prediction could be completed in under 5 min. For the phase II model selection: 10-fold cross-validation could be completed in approximately 20 min, and LOOCV cross-validation could be completed in less than 6 days. The optimizations for speed used did however, require a substantial investment of memory, and our analyses required at least 8–16 GB of RAM.

### Data simulation methods

For each simulated GWAS, different causal variants were randomly generated, with each causal SNP varying by minor allele frequency (selected at random from the MAF’s found on chromosome 1 of the Illumina Human 610-Quad beadchip, all MAF’s > 0.05), disease allele (A or B), effect strength (within the scenario-assigned bounds, from OR_min_ to OR_max_), and mode of inheritance (dominant, recessive, or additive). The total number of causal SNPs was constant: for simulation sets 1 and 2, each GWAS contained 50 pleiotropic SNPs associated with both *D*_A_ and *D*_B_, 50 SNPs associated only with *D*_A_, and 50 SNPs associated only with *D*_B_. For simulation set 3, each GWAS contained 75 SNPs associated only with *D*_A_, and 75 SNPs associated only with *D*_B_.

All non-causal SNPs were assumed to be independent of one another and of phenotype status. Minor allele frequencies were all above 0.05, and were selected from the Caucasian HapMap estimates for the Illumina Human 610-Quad beadchip. Genotype frequencies were calculated from minor allele frequencies to conform to Hardy–Weinberg equilibrium.

For each causal SNP, once MAF, disease allele, odds ratio, and mode of inheritance were selected, genotype frequencies were calculated for each phenotype class. For subject classes that did not have a disease status associated with a SNP [e.g., for a *D*_a_-associated SNP, this would be the subject classes $\vec{D}$ = (1,1) and $\vec{D}$ = (1,2)], genotype frequencies conformed to Hardy-Weinberg equilibrium. For subject classes that were associated with the SNP, genotype frequencies were transformed to yield the assigned odds ratio. For the allelic model, the assigned odds ratio was set as the odds ratio between the opposite homozygous genotypes (i.e., “AA” and “BB”), and the odds ratio for the “AA” and “AB” genotypes was set to the square root of the full OR parameter. For pleiotropic SNPs, the two effects were functionally additive.

All SNPs were assumed to be conditionally independent from one another, by phenotype status.

Subjects were first assigned phenotype values for *D*_A_ and *D*_B_, based on the counts set by the scenario, and then genotypes were randomly generated for each subject as a function of phenotype class, with each genotype being drawn from the 3-value discrete (or “categorical”) distribution with parameters set to the genotype frequencies for the subject’s assigned phenotype class.

### Real data analysis

Genetic and phenotypic data was collected from five different studies (See Table 9). These datasets, genotype data, and quality control procedures are described elsewhere (Milton et al., 2012). The first and largest of the five, the CSSCD, was used as the discovery set.

**Table 9:** **Summary of data sources**.

Dataset	No of genotyped subjects, after cleaning			
	Total	Total w/clean HbF reading	Stroke cases, total	Stroke cases, w/HbF reading
CSSCD	1071	778	83	63
MSH	140	140	9	9
PUSH	97	51	16	5
WP	45	44	3	3
C-Data	117	117	20	20

Four smaller study datasets were used for validation: the MSH, Pulmonary Hypertension and the Hypoxic Response in SCD (PUSH), Treatment of Pulmonary Hypertension and SCD with Sildenafil Treatment (walk-PHaSST, or WP), and the Comprehensive Sickle Cell Centers C-Data Project.

Some of the subjects in the PUSH, WP, and C-Data datasets were on hydroxyurea at the time of HbF measurement. Since hydroxyurea operates by increasing the production of HbF, and since it may have an effect on CVA, subjects on hydroxyurea were dropped.

For HbF, readings taken before age 5 were discarded, and the median of the remaining measurements were used. Since our methods are only designed to deal with dichotomous variables, HbF was dichotomized into high (≥ 8.6), and low (< 8.6) levels, as HbF above 8.6 has been shown to be associated with improved clinical prognosis (Okser et al., 2010). Missing HbF values were imputed using a regression model of HbF as a function of white blood cell count (WBC), mean corpuscular volume (MCV), hematocrit (HCT), age, and sex.

## Conflict of Interest Statement

The authors declare that the research was conducted in the absence of any commercial or financial relationships that could be construed as a potential conflict of interest.
